# Appointed to the U. S. Navy

**Published:** 1868-04

**Authors:** 


APPOINTED TO THE U. S. NAVY.—We are pleased to notice the appointment of Dr. P. P. Bielby, as Ass't Surgeon U. S. Navy. We understand that the examining board have been in session since January, and have examined over thirty candidates, of which number only five have passed. The rigid examination made by this board, and the high standard of attainment required makes it a real triumph when a young physician receives its approbation. Dr. Bielby was a member of the last graduating class in the Buffalo Medical College, and his numerous friends in Buffalo and vicinity will take much pleasure in this early recognition of his high merit.

